# Complicated adult right-sided Bochdalek hernia with Chilaiditi’s syndrome: a case report

**DOI:** 10.1186/s40792-015-0100-y

**Published:** 2015-10-06

**Authors:** Motonobu Watanabe, Osamu Ishibashi, Muneaki Watanabe, Tadashi Kondo, Nobuhiro Ohkohchi

**Affiliations:** Department of Surgery, Graduate School of Comprehensive Human Sciences, University of Tsukuba, 1-1-1 Tennoudai, Tsukuba, Ibaraki 305-8558 Japan; Department of Surgery, Mito Kyodo General Hospital, 3-2-7 Miyamachi, Mito, Ibaraki 310-0015 Japan; Department of Surgery, Moriya Daiichi General Hospital, 1-17 Matsumaedai, Moriya, Ibaraki 302-0102 Japan

**Keywords:** Bochdalek hernia, Chilaiditi's syndrome, Strangulated ileus, In adults

## Abstract

An extremely rare adult case that underwent surgery for ileus caused by Bochdalek hernia associated with Chilaiditi’s syndrome is presented. A 65-year-old woman complaining of upper abdominal pain presented to our hospital. Abdominal plain radiography showed increased intestinal gas, and computed tomography (CT) showed the transverse colon located above the right lobe of the liver, representing Chilaiditi’s sign. She was diagnosed as having ileus and treated with decompression therapy by a nasoenteric tube. After hospitalization, the patient developed dyspnea, and CT showed intestinal herniation into the right thoracic cavity. She was diagnosed as having strangulated ileus caused by Bochdalek hernia. An emergent laparotomy was performed, and it showed a hole of 5 cm in diameter at the right hemi-diaphragm. The transverse colon was incarcerated through the hole, it was pulled back to the abdominal cavity, and a right hemicolectomy was performed because of necrotic changes. A small part of the liver was also herniated into the right thoracic cavity, and it was returned to the abdominal cavity. The defect in the diaphragm was closed by direct suture. Although the patient developed an abscess in the thoracic cavity postoperatively, she improved with antibiotic therapy and was discharged 2 months after the operation.

## Background

Bochdalek hernia is one of the most common congenital diaphragmatic hernias, and most cases are diagnosed in the neonatal period with clinical features related to the respiratory system [1]. In contrast, symptoms are rare in adults, and it is usually incidentally discovered in patients with gastrointestinal symptoms [2]. Chilaiditi’s sign is also a rare radiographic finding of subdiaphragmatic radiolucency due to interposition of the intestine between the liver and the diaphragm, and when it is associated with digestive symptoms, it is termed Chilaiditi’s syndrome [3, 4]. In both Bochdalek hernia and Chilaiditi’s Syndrome, if the symptoms progress to bowel obstruction or ischemia, emergent surgical treatment is needed. In this paper, an extremely rare adult case that underwent surgery for ileus caused by a Bochdalek hernia associated with Chilaiditi’s syndrome is presented.

## Case presentation

**Fig. 1:**
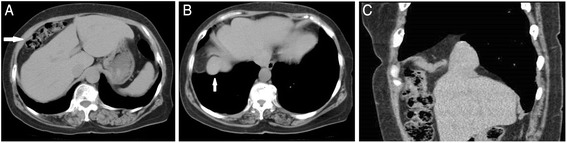
**a** Computed tomography (CT) of the abdomen showing the transverse colon located above the right lobe of the liver (*arrow*). **b** CT showing a small part of the liver herniated into the right thoracic cavity (*arrow*). **c** Sagittal view of the same CT showing a small part of the liver and intestinal fat tissue in the right thoracic cavity

**Fig. 2:**
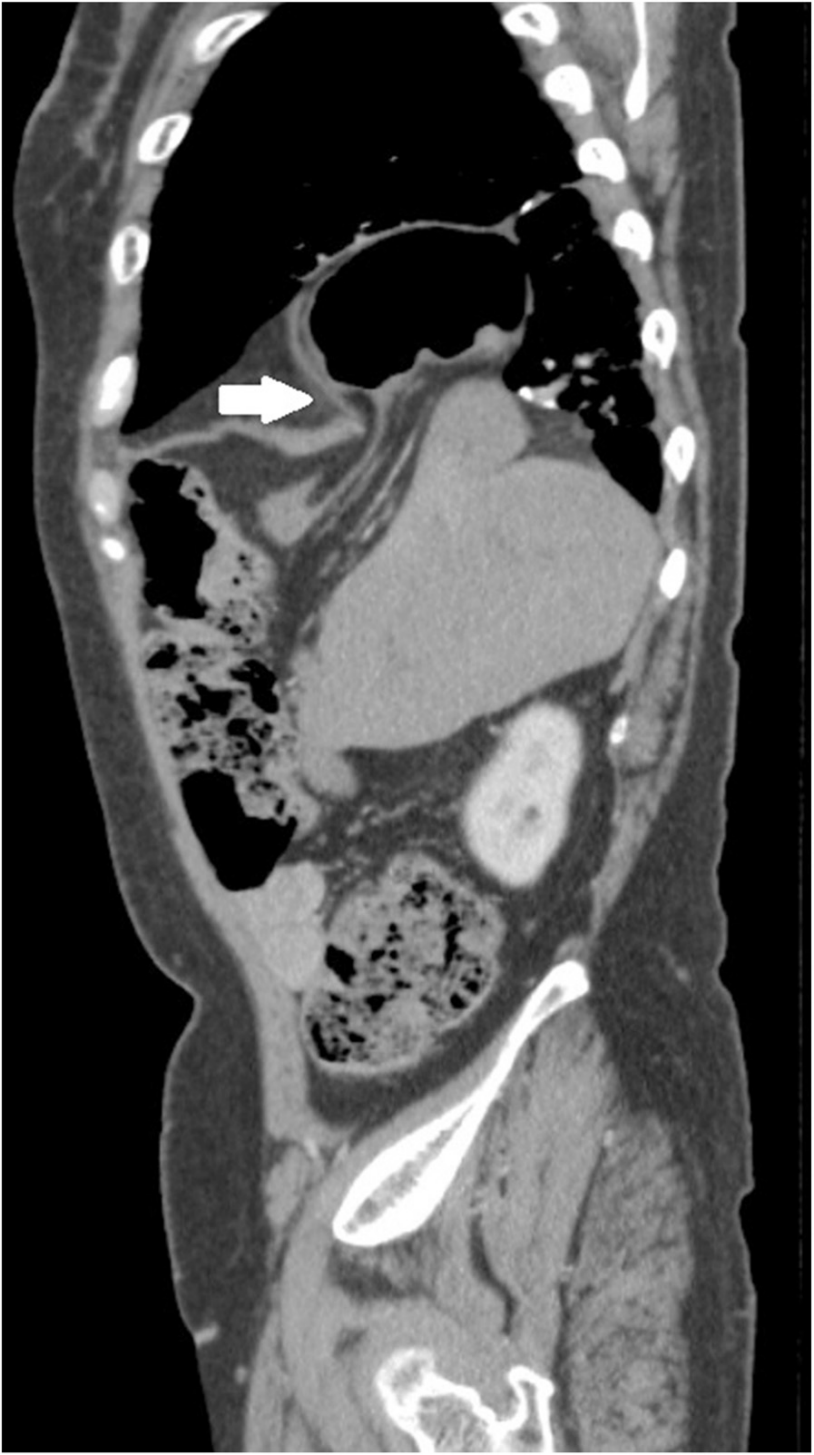
Sagittal sequence of CT shows intestinal incarceration in the right thoracic cavity through the hole through which a part of the liver has herniated (*arrow*)

A 65-year-old woman visited our hospital with a complaint of continuous upper abdominal pain. She had a medical history of rheumatoid arthritis and was on prednisolone 5 mg a day. On admission, she was afebrile and had no abdominal distention, but she had tenderness over the whole abdomen, and her bowel sounds were slightly decreased. Apart from minimal hypokalemia, her laboratory values were normal. Abdominal X-ray showed small intestinal gas and the computed tomography showed the transverse colon located above the right lobe of the liver, known as Chilaiditi’s sign (Fig. 1a). A small part of the liver and intestinal fat tissue had also been seen in the bottom of the right thoracic cavity, probably due to the congenital hernia (Fig. 1b, c). She was diagnosed as having ileus due to Chilaiditi’s syndrome and was treated with decompression therapy by a nasoenteric tube. Four days later, she developed dyspnea and her abdominal pain worsened. Laboratory examination showed elevations in the white blood cell count (10,900/μl), C-reactive protein (19.48 mg/dl), fibrin/fibrinogen degradation products (FDP) (12.1 μg/ml), aspartate aminotransferase (AST) (43 U/l), and alanine aminotransferase (ALT) (43 U/l). Chest-abdominal computed tomography (CT) showed intestinal incarceration in the right thoracic cavity (Fig. 2). With these findings, the patient was diagnosed as having incarceration due to a right Bochdalek hernia and Chilaiditi’s syndrome.

**Fig. 3:**
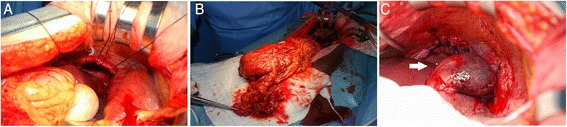
**a** Intraoperative photographs showing a hole of 5 cm in diameter at the right hemi-diaphragm. **b** The part of the transverse colon that was pulled back into the abdominal cavity from the right thoracic cavity. **c** A part of the right lobe of the liver that was returned to the abdominal cavity from the thoracic cavity (*arrow*). This part of the liver adhered to the bottom lobe of the right lung. It was separated from the lung and returned to the abdominal cavity

**Fig. 4:**
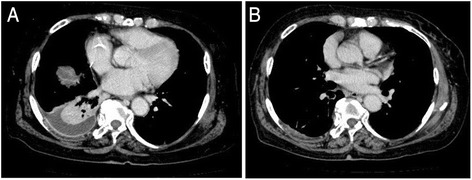
**a** Two-week postoperative chest-abdominal CT shows an abscess in the right thoracic cavity. **b** Six-week postoperative CT shows disappearance of the abscess in the right thoracic cavity

Exploratory laparotomy was performed, and it showed a hole of 5 cm in diameter at the right hemi-diaphragm (Fig. 3a). The transverse colon was herniated into the right thoracic cavity through the hole. A small part of the right lobe of the liver was also herniated into the cavity and adhered to the bottom lobe of the right lung. The transverse colon was pulled back into the abdominal cavity, but a right hemicolectomy was performed because the incarcerated part had developed necrosis and showed micro perforation (Fig. 3b). The herniated part of the liver was separated from the lung and returned to the abdominal cavity (Fig. 3c). The diaphragmatic defect was closed by direct suture with 1–0 absorbable thread, and drains were placed into the right thoracic cavity and right subphrenic space. The patient developed an abscess in the right thoracic cavity after removal of the chest drain postoperatively (Fig. 4a), and she was treated with systemic antibiotics. The abscess resolved with treatments (Fig. 4b), and she was discharged 2 months after the operation. As of 56 months after the surgery, she is doing well without recurrence of any symptoms.

### Discussion

Chilaiditi’s sign is a rare radiological finding of segmental interposition of the colon between the liver and the diaphragm. Chilaiditi’s syndrome refers to a clinically symptomatic patient with radiographic findings; the syndrome was first described by Demetrius Chilaiditi in 1910 [3]. Chilaiditi’s sign has been reported in association with various conditions, such as liver cirrhosis, segmental agenesis of the liver, relaxation of the suspensory ligament, and hypermobile colon with long mesentery, constipation, multiple pregnancies, obesity, and significant weight loss [4]. Although the condition is usually asymptomatic and found incidentally, it sometimes occurs with abdominal pain, distention, vomiting, constipation, and obstruction. Treatment is usually conservative, but if the symptoms progress to mechanical bowel obstruction and bowel ischemia, surgical treatment is needed [5, 6].

Congenital hernias of the diaphragm can be classified into four different types: eventration of the diaphragm, posterolateral hernia of Bochdalek, parasternal hernia of Morgagni-Larrey, and peritoneopericardial hernia [7]. A Bochdalek diaphragmatic hernia is one of the most common congenital diaphragmatic hernias in infants and can result in severe respiratory distress, necessitating immediate surgery. In contrast, its presentation in adulthood is rare, and it is usually discovered incidentally in patients presenting with gastrointestinal symptoms [2, 8]. Most of the hernias are found on the left side, and right-sided hernias are rarer because the right pleuroperitoneal canal closes earlier and the liver buttresses the right diaphragm [9]. Fewer than 200 cases of adult Bochdalek hernia have been reported, with even fewer right-sided hernias in adults [8]. Moreover, a literature search using the term ‘Bochdalek hernia’ in combination with ‘right-sided’ and ‘colon’ and ‘adult’ returned only 11 cases, in addition to the present case [1, 9–18].

In the present case, at the time of admission, abdominal CT showed the transverse colon located above the right lobe of the liver, representing Chilaiditi’s sign. Furthermore, intestinal fat tissue and a part of the liver were also herniated into the right thoracic cavity, probably due to the Bochdalek hernia. After hospitalization, the patient gradually developed dyspnea, and CT showed intestinal herniation into the right thoracic cavity. These findings indicate that the increased intraperitoneal pressure due to ileus induced incarceration of the colon into the thoracic cavity through the defect next to the herniated liver. Although an emergent laparotomy was performed, a right hemicolectomy was needed because of necrotic changes.

Most Bochdalek hernias are treated surgically with thoracotomy or laparotomy, and several cases of thoracoscopic and laparoscopic repairs have recently been reported. The transthoracic approach enables direct observation of the herniated viscera, and the hilum of the hernia or sac, and allows easy removal of the herniated viscera. The transperitoneal approach also allows confirmation of the position of the viscera and easy repairs [2]. However, in difficult cases such as the present case, the open transperitoneal approach is preferable to achieve emergent release of the incarceration and repair of the damaged viscera.

The diaphragmatic defect is repaired by primary closure or with a prosthetic mesh or muscle flap. Primary repair is performed when there is sufficient diaphragm to close without tension, and non-absorbable thread is usually used to close the defect to avoid recurrence. Prosthetic mesh has been used to repair large congenital diaphragmatic hernias, but it should not be used in contaminated situations [8, 19]. In the present patient, there was sufficient diaphragm for direct closure without tension, and absorbable thread was used to minimize the risk of infection postoperatively as it was a contaminated case. The patient has had no recurrence of hernia for 56 months after the operation, and further follow-up is needed.

## Conclusions

An extremely rare case of a right-sided Bochdalek hernia associated with Chilaiditi’s syndrome in an adult who was treated via laparotomy was described. Chilaiditi’s sign and Bochdalek hernia are usually asymptomatic, and the disorder may manifest eventually. Symptoms and clinical status may vary from mild to severe, so clinicians should consider this disorder in their daily examinations and treat such cases appropriately to avoid complications.

## Consent

Written informed consent was obtained from the patient for publication of this case report and accompanying images. A copy of the written consent is available for review by the Editor-in-Chief of this journal.

## Abbreviations

ALT: alanine aminotransferase
AST: aspartate aminotransferase
CT: computed tomography
FDP: fibrin/fibrinogen degradation products
